# Botulinum Toxin Effects on Sensorimotor Integration in Focal Dystonias

**DOI:** 10.3390/toxins12050277

**Published:** 2020-04-25

**Authors:** Maria Ilenia De Bartolo, Nicoletta Manzo, Gina Ferrazzano, Viola Baione, Daniele Belvisi, Giovanni Fabbrini, Alfredo Berardelli, Antonella Conte

**Affiliations:** 1 IRCCS NEUROMED, Via Atinense, 18, 86077 Pozzilli (IS), Italy; mariailenia.debartolo@uniroma1.it (M.I.D.B.); nicoletta.manzo@uniroma1.it (N.M.); daniele.belvisi@uniroma1.it (D.B.); giovanni.fabbrini@uniroma1.it (G.F.); antonella.conte@uniroma1.it (A.C.); 2 Department of Human Neuroscience, Sapienza University of Rome, 00185 Rome, Italy; gina.ferrazzano@uniroma1.it (G.F.); viola.baione@uniroma1.it (V.B.)

**Keywords:** sensorimotor integration, somatosensory temporal discrimination threshold, focal dystonia, botulinum toxin A, cervical dystonia, focal hand dystonia, blepharospasm

## Abstract

(1) Background: In dystonia, the somatosensory temporal discrimination threshold (STDT) is abnormally increased at rest and higher and longer-lasting during movement execution in comparison with healthy subjects (HS), suggesting an abnormal sensorimotor integration. These abnormalities are thought to depend on abnormal proprioceptive input coming from dystonic muscles. Since Botulinum toxin-A (BT-A) reduces proprioceptive input in the injected muscles, our study investigated the effects of BT-A on STDT tested at rest and during voluntary movement execution in patients with focal dystonia. (2) Methods: We enrolled 35 patients with focal dystonia: 14 patients with cervical dystonia (CD), 11 patients with blepharospasm (BSP), and 10 patients with focal hand dystonia (FHD); and 12 age-matched HS. STDT tested by delivering paired stimuli was measured in all subjects at rest and during index finger abductions. (3) Results: Patients with dystonia had higher STDT values at rest and during movement execution than HS. While BT-A did not modify STDT at rest, it reduced the abnormal values of STDT during movement in CD and FHD patients, but not in BSP patients. (4) Conclusions: BT-A improved abnormal sensorimotor integration in CD and FHD, most likely by decreasing the overflow of proprioceptive signaling from muscle dystonic activity to the thalamus.

## 1. Introduction

Temporal discrimination can be tested in humans with the somatosensory temporal discrimination threshold (STDT) technique, which assesses the interval needed to recognize a pair of stimuli as separate in time [1]. Experimental studies performed in healthy subjects (HS) have shown that STDT is decoded by a cortical-subcortical loop involving sensory motor cortex (S1) and the basal ganglia [2,3]. In HS, STDT values increase when tested during voluntary movement execution and return to normal after the end of the movement. The increase in STDT during movement execution reflects mechanisms of sensorimotor integration, specifically the process of gating tactile input during movement. This gating is necessary in order to prioritize proprioceptive input. Several studies have concluded that sensorimotor integration between tactile stimuli and voluntary movement is influenced by central mechanisms, including thalamo-basal ganglia interplay [4,5]. In dystonia, a movement disorder that involves altered mechanisms of sensorimotor integration [2,6,7,8], STDT tested at rest is abnormally higher [2]. In contrast to HS, patients with dystonia also show higher and longer lasting STDT modulation during movement execution [5], again confirming abnormalities in the sensorimotor processes.

Botulinum toxin A (BT-A), the gold standard therapy for focal dystonia [9,10], improves dystonia by reducing excessive muscle contractions through the inhibition of acetylcholine release from the presynaptic membrane. Several studies have also shown that BT-A induces changes in proprioceptive afferent input processing in dystonia patients by also modulating gamma innervation and spindle tuning in dystonic muscles [11,12,13,14]. Only one study, which was performed in our laboratory, tested the effect of BT-A on STDT in dystonia. We found that BT-A did not modify abnormal STDT values tested at rest in cervical dystonia (CD) patients [15]. However, it is unknown whether in dystonia BT-A injected in the dystonic muscles has an effect on STDT values when tested during movement execution. We hypothesized that in dystonia BT-A injection into affected muscles reduces proprioceptive input and determines changes in central mechanisms of sensorimotor integration, thus ameliorating abnormal STDT modulation during movement execution. To verify this hypothesis, we tested STDT modulation during index finger abduction in dystonia patients who underwent BT-A injection into muscles that were either close to or remote from the body district involved in the motor task.

The aim of the present study was to investigate whether BT-A influences STDT modulation induced by movement execution in patients with dystonia. In order to do so, we tested STDT during voluntary movement execution in HS and in patients with focal dystonia before and 1 month after treatment with BT-A. Investigating how BT-A modifies STDT modulation in patients with focal dystonia could help clarify the role of proprioception and sensorimotor integration in the pathophysiology of different types of focal dystonia.

## 2. Results

Before BT-A, STDT values tested at rest were higher in dystonia patients than in HS (*p* = 0.01). STDT values were similar in the three dystonia patient groups (Table 1) STDT values tested at rest remained unchanged after BT-A injection in the three patient groups studied (all *p* > 0.05).

Between-group ANOVA comparing STDT changes during movement execution in patients before BT-A and HS disclosed significant factors ‘GROUP’ (F = 6.66, *p* = 0.001) and ‘ISI’, where ISI is considered the interstimulus interval between movement onset and the deliverance of electrical stimuli (F = 52.9, *p* < 0.001), and a significant ‘GROUP × ISI’ interaction (F = 2.75, *p* = 0.006). Between-group ANOVA comparing STDT changes during movement execution in patients after BT-A therapy and HS disclosed significant factors ‘GROUP’ (F = 2.7, *p* = 0.05) and ‘ISI’ (F = 54.1, *p* < 0.001) and a significant ‘GROUP × ISI’ interaction (F = 3.33, *p* = 0.001).

Repeated measures ANOVA evaluating changes in STDT values during movement execution before and 1 month after BT-A injection in CD patients showed significant factors ‘BT-A’ (F = 18.0, *p* < 0.01) and ‘ISI’ (F = 19.5, *p* < 0.001) and a significant ‘BT-A x ISI’ interaction (F = 11.8, *p* < 0.001). Similarly, in FHD patients, analysis disclosed a significant factor ‘ISI’ (F = 16.5, *p* < 0.001), a significant ‘BT-A x ISI’ interaction (F = 3.0, *p* = 0.04), and a trend towards a significant factor ‘BT-A’ (F = 3.8, *p* = 0.07).

**Figure 1:**
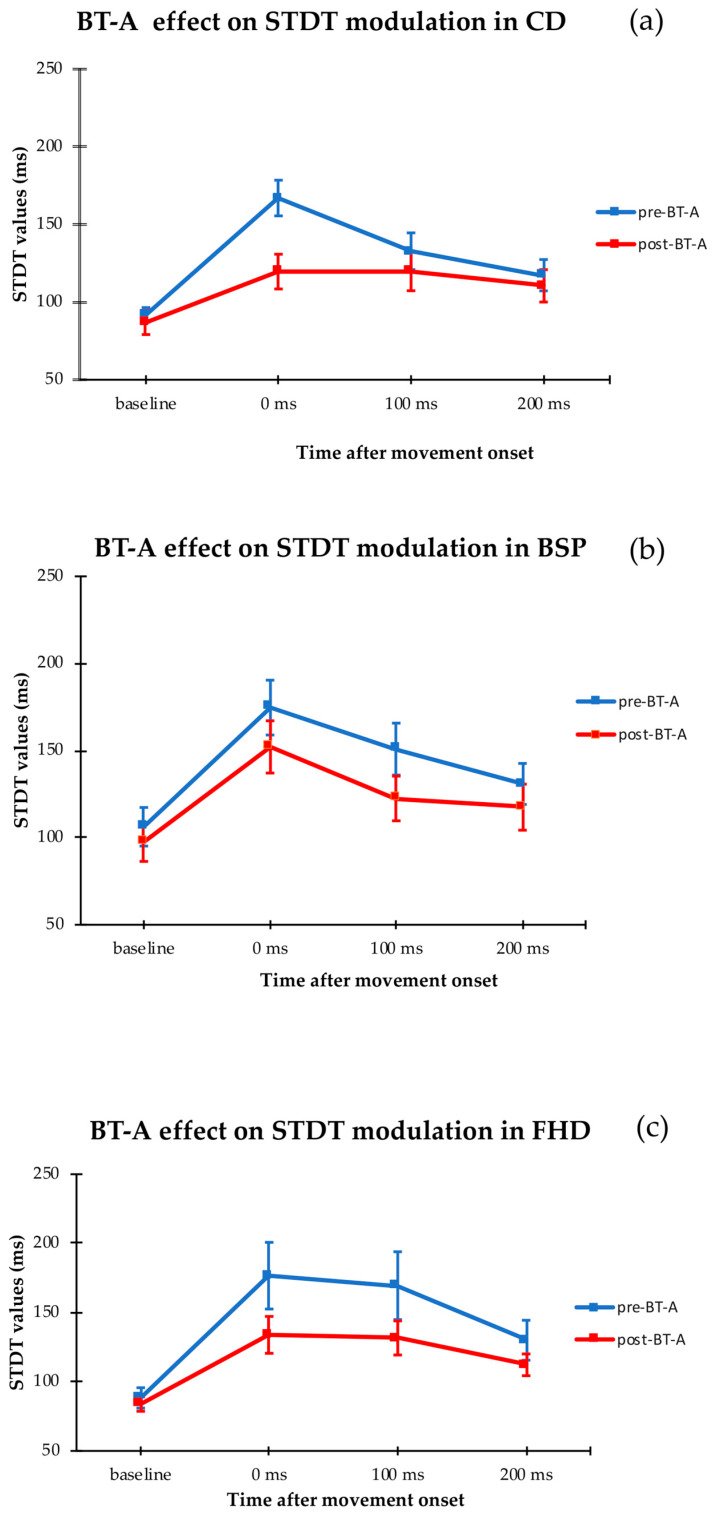
Somatosensory temporal discrimination threshold (STDT) values at rest and at 0, 100 and 200 ms after movement onset, before (blue line) and after (red line) BT-A treatment in cervical dystonia (CD) (**a**), blepharospasm (BSP) (**b**) and focal hand dystonia (FHD) patients (**c**).

Repeated measures ANOVA evaluating changes in STDT values during movement execution before and 1 month after BT-A injection in BSP patients showed a significant factor ‘ISI’ (F = 18.6, *p* < 0.001), but no significant factor ‘BT-A’ (F = 2.8, *p* = 0.12) or ‘BT-A x ISI’ interaction (F = 2.0, *p* = 0.13). Therefore, BT-A injection changed STDT modulation during index finger movement in CD and FHD patients, but not in BSP patients (Table 1; Figure 1).

Repeated-measures ANOVA evaluating the kinematic features of index finger movements showed no significant factors ‘BT-A’ or ‘ISI’, nor any significant interactions in the three patient groups (all *p* > 0.05).

The duration of BT-A treatment did not differ in the three groups of patients (F = 0.19; *p* = 0.82).

BT-A treatment improved disease severity scale scores of dystonic muscle spasms in the three patient groups (CD patients: *p* < 0.001; BSP: *p* < 0.001; FHD: *p* = 0.03).

Pearson’s correlation coefficient did not disclose any significant relationship between clinical severity score changes and the extent of BT-A-induced STDT modulation changes (all *p* > 0.05).

## 3. Discussion

In the present paper we confirmed that STDT baseline values were higher in focal dystonia when compared with HS [2]. BT-A did not modify STDT values tested at rest in BSP, FHD, or CD patients [15]. The novel finding of our study is that BT-A significantly reduced the abnormally higher STDT modulation during movement execution in CD and FHD patients, but not in BSP patients. This implies that sensorimotor integration, which is abnormal in dystonia [2,5], can be improved by BT-A in CD and FHD patients.

To exclude the possibility that STDT changes during movement were related to different levels of attention, the trial order, characterized by different intervals between movement onset and paired stimuli, was randomized. The improvement in STDT modulation after BT-A was not due to learning effect related to the repetition of the task (before and after BT-A injection) because we pseudorandomized the order of sessions before and after BT-A injections. We also randomized the different trials (0 ms, 100 ms, 200 ms) in each participant. If the improvement in STDT modulation after BT-A was due to a learning effect, we should have observed the same extent of improvement in the three groups. Conversely, STDT modulation before and after BT-A did not significantly change in BSP patients. Finally, previous studies in healthy subjects investigating STDT gating during movement [4,16] showed that STDT modulation tested twice in the same subjects did not vary between the two sessions. These results further exclude the possibility that the effects induced by BT-A in dystonic patients are related to learning mechanisms. We also used the same brand of BT-A for the three patient groups to exclude possible differences in clinical and neurophysiological outcomes related to different pharmacokinetic properties of various BT-A preparations. Finally, to evaluate whether STDT changes during movement execution were related to different motor performance among patients with different types of focal dystonia and HS, we recorded kinematic parameters of index finger movement. Consistent with previous findings [5], we found no difference in motor performance across groups.

The novel finding of the present study is that BT-A improves sensorimotor integration as tested by STDT modulation in CD and FHD patients. In HS we have previously demonstrated that STDT increases when tested during voluntary movement execution and that the increase is maximal at movement onset and returns to normal values at the end of the movement [4,17]. Similar results have also been reported by Lei et al. [16]. In HS, the STDT increase observed during movement execution may be due to the interference that voluntary movement determines on the central processing of tactile input. We previously found, and have now confirmed, that STDT increases during movement to a larger extent and with a longer duration in CD and FHD patients as compared with HS [5]. We theorize that in CD and FHD patients the excessive proprioceptive input from dystonic muscle spindle afferents competes with tactile-input processing at the thalamic level, adding “noise” to sensorimotor integration mechanisms. The present finding that BT-A injected into dystonic muscles improves sensorimotor integration (as demonstrated by the reduced STDT modulation during movement) in body parts that are both affected (FHD) and unaffected (CD) by dystonia suggests that, by decreasing proprioceptive input directed to the thalamus, BT-A may ameliorate central mechanisms of sensorimotor integration, even those related to body parts unaffected by dystonia. The extent of BT-A effect on STDT modulation during movement appears to be more evident if the BT-A is injected in muscles close to those involved in movement execution. Several lines of evidence are consistent with our hypothesis. BT-A restored motor map representations in CD and FHD patients [18,19], normalized the abnormally higher P22/N30 cortical component of somatosensory evoked potentials in CD patients [20], and transiently modified the excitability of the cortical motor areas by reorganizing inhibitory and excitatory intracortical circuits in FHD patients [14]. More recently, Khosravani et al. (2020) have demonstrated that BT-A normalized the cortical processing of proprioceptive information from non-symptomatic limbs in CD patients [21]. Furthermore, a central indirect effect of BT-A in CD has been strongly suggested by functional MRI (fMRI) studies in which BT-A treatment resulted in a partial restoration of connectivity abnormalities in the sensorimotor networks [22,23,24].

BT-A-induced improvement in STDT modulation in CD and FHD patients did not correlate with motor symptom improvement as clinically tested using standardized clinical scales. It is known that clinical motor improvement induced by BT-A in dystonia is mostly dependent on its effect on extrafusal fibers [9], while STDT modulation improvement after BT-A is likely due to its effect on intrafusal fibers, which is clinically less relevant than motor improvement. The clinical correlate of abnormal sensorimotor integration is likely not dystonic activity severity, but more complex behavioral patterns of motor performance, such as movement accuracy.

In the present study, we observed that the kinematic parameters of index finger abduction were normal in patients affected by CD, BSP, and FHD. Previous studies reported conflicting results regarding movement performance in dystonia patients [25,26,27,28]. While it was to be expected that hand movement performance was normal in BSP and CD patients, it is more difficult to explain this finding in FHD patients. A possible reason is that our task did not induce dystonic movements in our FHD patients. Indeed, we asked patients to perform a fast index finger abduction with no forearm muscle involvement. However, focal dystonia in our patients mainly involved carpus and finger flexors and extensors, which were not activated by our task. In line with this hypothesis, previous studies using the same task reported a normal motor performance in FHD patients [5,26]. One interesting question concerns the clinical correlate of abnormal sensorimotor integration in patients with dystonia.

In conclusion, we have provided evidence that BT-A improves abnormal sensorimotor integration in CD and FHD patients, as tested by STDT movement-induced modulation. We believe that the BT-A-induced improvement depends on BT-A’s indirect central effects, which modulate proprioceptive overflow from dystonic muscles to the thalamus. Concordantly, BT-A treatment did not influence STDT modulation during movement in BSP patients, who conceivably have negligible proprioceptive overflow related to the dystonic ocular muscle as compared with CD and FHD patients [5]. Our study gives further insight into the role of altered sensorimotor integration as a pathophysiological mechanism involved in focal dystonia. Future neurophysiological studies specifically designed to assess biomechanical properties of voluntary movement in dystonia are needed to clarify the behavioral correlate of abnormal sensorimotor integration in focal dystonia.

## 4. Materials and Methods

### 4.1. Subjects

Thirty-five patients with different types of focal dystonia were enrolled, including 14 patients with CD, aged 54 ± 9 years (Table 2); 11 patients with BSP, aged 59 ± 8 years (Table 3); and 10 patients with FHD, aged 47 ± 16 years (Table 4). These patients were compared to 12 age-matched HS. All patients were recruited from the movement disorders outpatient clinic at the Department of Human Neurosciences, Sapienza University of Rome. Diagnosis of focal dystonia was made by a neurologist experienced in movement disorders and based on current clinical criteria [29]. Disease severity was evaluated in BSP patients using the Blepharospasm Severity Rating Scale (BSRS) [30] (Table 3), in CD patients using the Toronto Western Spasmodic Torticollis Rating Scale (TWSTRS) (Table 2), and in FHD patients using the Burke-Fahn-Marsden Dystonia Rating Scale (Table 4).

All patients underwent the experimental procedure and the clinical examination twice: 1 month after BT-A injection (post-BT-A) (incobotulinum toxin A) [Xeomin^®^, Merz] and after a BT-A wash-out period at least 3-4 months after the last BT-A injection (pre-BT-A). The order of the two sessions (pre-BT-A and post-BT-A) was pseudorandomized across individuals, except for three FHD patients who performed the pre-BT-A evaluation first. The timeframe of 3-4 months after the last BT-A treatment was established in each patient according to his/her own clinical history, previous response to BT-A, and the frequency of injections. To ensure the reliability of STDT testing, patients with a Mini-Mental State Examination (MMSE) score lower than 26 and those with clinical signs of peripheral neuropathy were excluded. The study was approved by the institutional review board Sapienza University of Rome Ethics Committee (approval code: 4041, approval date: 24/03/2016) and the experimental procedure was conducted in accordance with the Declaration of Helsinki. All participants gave their written informed consent.

### 4.2. STDT Testing

To assess STDT values we applied the experimental protocol used in previous studies [4,31,32,33]. With patients comfortably seated in an armchair, we used a constant current stimulator (Digitimer DS7AH) to deliver paired tactile stimuli. Stimuli consisted of 0.1 ms square-wave electrical pulses delivered through surface skin electrodes (1 mm diameter) with the anode located 0.5 cm distally to the cathode. Surface skin electrodes were applied to the volar surface of the distal phalanx of the right index finger in HS and BSP and CD patients, and to the volar surface of the distal phalanx of the index finger on the affected side in FHD patients. Stimulation intensity was defined by delivering a series of stimuli at increasing intensities starting at 2 mA and increasing in steps of 0.5 mA. The intensity varied in each subject depending on the minimal intensity needed by the subject to perceive 10 out of 10 consecutive stimuli. Paired stimuli were delivered starting from an interstimulus interval of 0 ms (simultaneous pair) and progressively increasing by 10 ms intervals using a staircase method. STDT was considered the first of three consecutive interstimulus interval at which subjects recognized the stimuli as temporally separate in an average of three STDT trials. A single trial was used as a catch trial to check possible changes in attention levels when ISI was higher than that corresponding to the threshold.

### 4.3. Electromyographic Recordings

Electromyographic (EMG) activity was recorded through a pair of surface (Ag/AgCl) cup electrodes placed over the first dorsal interosseous (FDI) muscle in a belly-tendon configuration. EMG signals were recorded and filtered with Digitimer D360 (Digitimer Ltd., Welwyn Garden City, Hertfordshire, UK) (bandwidth 20 Hz-1 kHz), then analyzed offline with a personal computer through a 1401-plus A/D laboratory interface (Cambridge Electronic Design, UK). Data were stored on a laboratory computer for online visual display and further offline analysis (Signal software; Cambridge Electronic Design). EMG activity from the FDI muscle during voluntary movement was measured by assessing the root mean square (RMS) amplitude. Electromyographic recordings were used to trigger the stimuli.

### 4.4. Kinematic Recordings

The SMART analyzer motion system (BTS Engineering, Milan, Italy), equipped with three infrared cameras (sampling rate 120 Hz), was used to record index finger abductions. The arm was abducted at the shoulder by about 45–50˚, and the elbow joint was flexed at about 90˚. We used four optical markers to record finger abduction: one marker was placed over the distal phalanx of the index finger, another one was placed on the first metacarpophalangeal joint, and two were distributed over the first and fifth metacarpal, respectively. After a verbal “go” signal, subjects abducted the index finger, then returned the finger to the starting position. The movement was performed as rapidly and widely as possible. Marker displacements were reconstructed via a dedicated software program that ran an automatic algorithm to compute the range of motion (ROM), which represented the displacement of the index finger around its metacarpophalangeal joint expressed as the degree of the angle and mean velocity (degrees per second) (BTS Engineering, Milan, Italy).

### 4.5. Experimental Paradigm

First, we measured STDT at rest (i.e., in the absence of movement) and then during index finger abduction on the volar surface of the right index finger in each subject. In patients with FHD we tested STDT on the affected hand. Patients were asked to abduct the index finger as widely and rapidly as possible. We first recorded 10 index finger abductions without delivering any paired stimuli before starting the experimental procedure. Experimental tasks consisted of index finger abductions, with paired stimuli for STDT triggered at 0, 100, or 200 ms after movement onset. Movement threshold was set at 100 μV of EMG activity (defined as “0 ms” for simplicity). To define the time course of movement-induced STDT changes, paired stimuli were delivered as soon as EMG signals reached an amplitude of 100 μV (defined as “0 ms” for simplicity), 100 and 200 ms after movement onset. STDT values were thus calculated for each time lapse (0, 100, and 200 ms). At each index finger abduction, the interstimulus interval for STDT testing was progressively increased in 10-ms intervals until the subject recognized the two stimuli as sequential (10). The order of sessions for each time lapse was pseudorandomized. All participants were blinded to the time-lapse condition.

### 4.6. Statistical Analysis

Separate repeated measures analyses of variance (rmANOVAs) were performed to evaluate STDT changes in the three patient groups before and after BT-A. Separate between-group rmANOVAs with the factor ‘ISI’ were performed to evaluate any differences in STDT modulation between patient groups before and after BT-A and HS.

We also investigated possible correlations between changes in clinical scale values (post-BT-A/pre-BT-A ratio) and percentage changes in STDT modulation after BT-A (Pearson’s correlation coefficient). A *p* value < 0.05 indicated statistical significance.

## Figures and Tables

**Table 1:** Somatosensory temporal discrimination threshold (STDT) values at rest and at 0, 100, and 200 ms after movement onset in cervical dystonia (CD), blepharospasm (BSP), and focal hand dystonia (FHD) patients pre- and post-BT-A treatment and healthy subjects (HS). STDT values at rest and at 0, 100, and 200 ms after movement onset are expressed as the mean of the STDT values assessed in the three different sessions, while the average change is expressed as the % of STDT change assessed at the three intervals after movement onset. Note that the % change in STDT modulation during movement induced by BT-A is slightly, although not significantly, larger in FHD in comparison to CD.

Subjects	Pre-BT-A					Post-BT-A				
	Rest	0 ms	100 ms	200 ms	Average % Change	Rest	0 ms	100 ms	200 ms	Average % Change
**CD**	91 ± 5	167 ± 11	132 ± 12	118 ± 10	140 ± 9	86 ± 8	120 ± 11	119 ± 12	111 ± 11	128 ± 10
**FHD**	88 ± 8	176 ± 24	169 ± 25	130 ± 14	164 ± 10	84 ± 5	134 ± 13	131 ± 12	112 ± 8	141 ± 7
**BPS**	106 ± 11	175 ± 15	151 ± 14	130 ± 11	132 ± 8	97 ± 11	161 ± 15	122 ± 13	118 ± 13	127 ± 4
**HS**	74 ± 4	107 ± 5	88 ± 5	78 ± 4	118 ± 5	-	-	-	-	-

**Table 2:** Cervical dystonia (CD) patient demographic and clinical information.

PZ	Gender	Age (yrs)	Disease Duration (yrs)	BT-Atreatment Duration (yrs)	Muscles Injected with BT-A	TWSTRS Off	TWSTRS On
1	M	45	26	25	SPL	8	8
2	F	58	16	11	SPL	14	12
3	F	45	9	3	SPL	13	10
4	F	36	4	4	SCM, SPL	14	8
5	F	46	2	2	SCM, SPL, TPZ	16	12
6	F	45	4	3	SCM, SPL	13	9
7	F	55	13	12	SCM, TPZ	7	6
8	F	60	11	8	SCM, SPL	10	10
9	F	65	10	4	SPL	14	10
10	F	60	20	11	SPL	14	12
11	F	57	20	13	SPL	7	7
12	F	64	5	3	SPL	14	10
13	M	56	4	5	SPL	13	9
14	F	57	3	1	SCM, SPL, TPZ	15	12
**Avg**	F/M = 12/2	54 ± 9	11 ± 8	8 ± 6		12 ± 3	9 ± 2

F = female; M = male; SPL = splenius; SCM = sternocleidomastoid; TPZ = trapezius; TWSTRS= *Toronto* Western Spasmodic Torticollis *Rating Scale*; Off = at least 3/4 months after the last BT-A injection; On = approximately 1 month after the last BT-A injection.

**Table 3:** Blepharospasm (BSP) patient demographic and clinical information.

Pz	Gender	Age (yrs)	Disease Duration (yrs)	BT-A Treatment Duration (yrs)	Muscles Injected with BT-A	BSRS Off	BSRS On
1	F	71	8	8	OO	13	12
2	F	68	10	3	OO	8	6
3	F	65	8	7	OO, Pretarsal	6	2
4	F	54	4	3	OO, Pretarsal	13	12
5	F	47	8	4	OO	8	6
6	F	50	9	9	OO	6	2
7	M	66	9	4	OO, Pretarsal	14	12
8	F	55	8	3	OO, Pretarsal	6	6
9	M	54	5	3	OO	8	6
10	M	64	13	10	OO, Pretarsal	8	8
11	M	60	9	6	OO, Pretarsal	11	9
**Avg**	F/M = 7/4	59 ± 8	8 ± 2	5 ± 3		9 ± 3	7 ± 3

F = female; M = male; OO = orbicularis oculi; BSRS = Blepharospasm Severity Rating Scale; Off = at least 3/4 months after the last BT-A injection; On = approximately 1 month after the last BT-A injection.

**Table 4:** Focal hand dystonia patient demographic and clinical information.

Pz	Gender	Age (ys)	Side Affected	Duration Disease (yrs)	BT-A Treatment Duration (yrs)	Muscles Injected with BT-A	BFMOff	BFM On
1	M	52	R	2	1	FCR	4	3
2	M	49	L	25	5	FDS	2	2
3	M	58	R	22	18	FCR, FCU	6	4
4	M	59	R	23	9	FDP, FDS	4	2
5	F	66	R	25	23	FCR, FCU	5	4
6	F	40	L	1	<1	FCR, FCU	2	2
7	F	50	L	8	5	FDP, FDS	5	4
8	F	18	R	1	<1	FCU	2	2
9	F	22	R	1	<1	FCR, FCU	12	10
10	F	52	R	8	8	ECR	6	4
**Avg**	F/M = 6/4	47 ± 16	R/L = 7/10	12 ± 11			5 ± 3	4 ± 2

F = female; M = male; R = right; L = left; FCR = flexor carpi radialis; FDS = flexor digitorum superficialis; FCU = flexor carpi ulnaris; FDP = flexor digitorum profundus; ECR = extensor carpi radialis; BFM = Burke-Fahn-Marsden Dystonia Rating Scale; Off = at least 3/4 months after the last BT-A injection; On = approximately 1 month after the last BT-A injection. All FHD patients were right-handed.
